# Navigating the Sotatercept landscape: A meta‐analysis of clinical outcomes

**DOI:** 10.1002/clc.24173

**Published:** 2023-10-11

**Authors:** Amir Nasrollahizadeh, Hamidreza Soleimani, Ali Nasrollahizadeh, Seyedeh Melika Hashemi, Kaveh Hosseini

**Affiliations:** ^1^ Cardiac Primary Prevention Research Center, Cardiovascular Diseases Research Institute Tehran University of Medical Sciences Tehran Iran; ^2^ Tehran Heart Center, Cardiovascular Diseases Research Institute Tehran University of Medical Sciences Tehran Iran; ^3^ Non‐Communicable Diseases Research Center, Endocrinology and Metabolism Population Sciences Institute Tehran University of Medical Sciences Tehran Iran

**Keywords:** hemoglobin, pulmonary hypertension, Sotatercept, vascular resistance, WHO functional class

## Abstract

Pulmonary arterial hypertension (PAH) is a widespread condition that affects around 1% of the global population, with a higher prevalence among older individuals. The approach to managing PAH has undergone significant changes, requiring extensive treatment strategies. Sotatercept, an FDA‐approved medication, has recently attracted attention for its potential role in PAH therapy. However, information on its safety and effectiveness is scarce. In this study, we performed a meta‐analysis of existing randomized clinical trials to assess the impact of Sotatercept on PAH patients. Our findings revealed that those treated with Sotatercept showed greater improvement in pulmonary vascular resistance and World Health Organization functional class compared with placebo recipients. The occurrence of adverse events was similar between both groups. Importantly, the Sotatercept group displayed a considerably higher number of cases with an increase in hemoglobin levels. Considering that about 33% of PAH patients experience anemia and both anemia and polycythemia can adversely affect disease prognosis, additional research is necessary to establish the potential advantages and disadvantages of Sotatercept as a treatment choice, specifically regarding its erythropoietic properties.

## Graphical abstract

In this study, we performed a meta‐analysis on two randomized clinical trials to assess the impact of Sotatercept on PAH patients. The included papulation were 429 patients with PAH, of which 237 received Sotatercept and 192 received placebo. They were followed for 6 months.

Our findings revealed that those treated with Sotatercept showed greater improvement in pulmonary vascular resistance, right atrial pressure, mean pulmonary artery pressure, change in N‐terminal pro–B‐type natriuretic peptide, and World Health Organization functional class compared with placebo recipients.

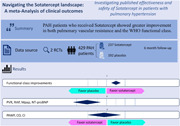

## 1 INTRODUCTION

Pulmonary arterial hypertension (PAH) affects roughly 1% of the global population, with a higher prevalence among older individuals.^1^ Furthermore, the approach to managing PAH patients has significantly evolved, necessitating a thorough treatment plan.^1^, ^2^ One FDA‐approved drug that has recently gained attention is Sotatercept. Sotatercept as a recombinant ActRIIA‐Fc fusion protein, binds to various activin‐class ligands. The anti‐inflammatory properties of ActRIIA‐Fc, along with its established ability to inhibit vascular remodeling, may contribute to the clinical effectiveness of Sotatercept as a treatment for PAH.^3^ Data assessing Sotatercept in regard to safety and efficacy are limited. To address this knowledge gap, we conducted a systematic review and meta‐analysis of published literature on Sotatercept.

## 2 METHODS

We conducted a search of several databases, including PubMed, EMBASE, Cochrane Library, Clinicaltrials.gov, and WHO's International Clinical Trials Registry Platform, from January 2009 to May 2023. We aimed to identify randomized controlled trials (RCTs) that reported on the effectiveness and adverse events associated with Sotatercept in treating PAH. The search strategy available in Supporting Information: Table 1 and the study protocol details were registered in the International Prospective Register of Systematic Reviews (PROSPERO)^4^ (PROSPERO ID: CRD42023429532).^5^ No language restriction was applied. A reference list of eligible studies and relevant reviews was also screened. The Risk of Bias Assessment Tool for Randomized Trials (RoB2) was used for appraising our included RCTs. All statistical analyses were conducted with R programming language (R for Windows, version 4.1.3), R Studio version 1.1.463 (Posit PBC) utilizing the “tidyverse” and “meta” statistical packages. For binary variables, risk ratios with 95% confidence intervals were calculated. For continuous variables, mean and standard deviation (SD) were calculated.

## 3 RESULTS

**Figure 1:**
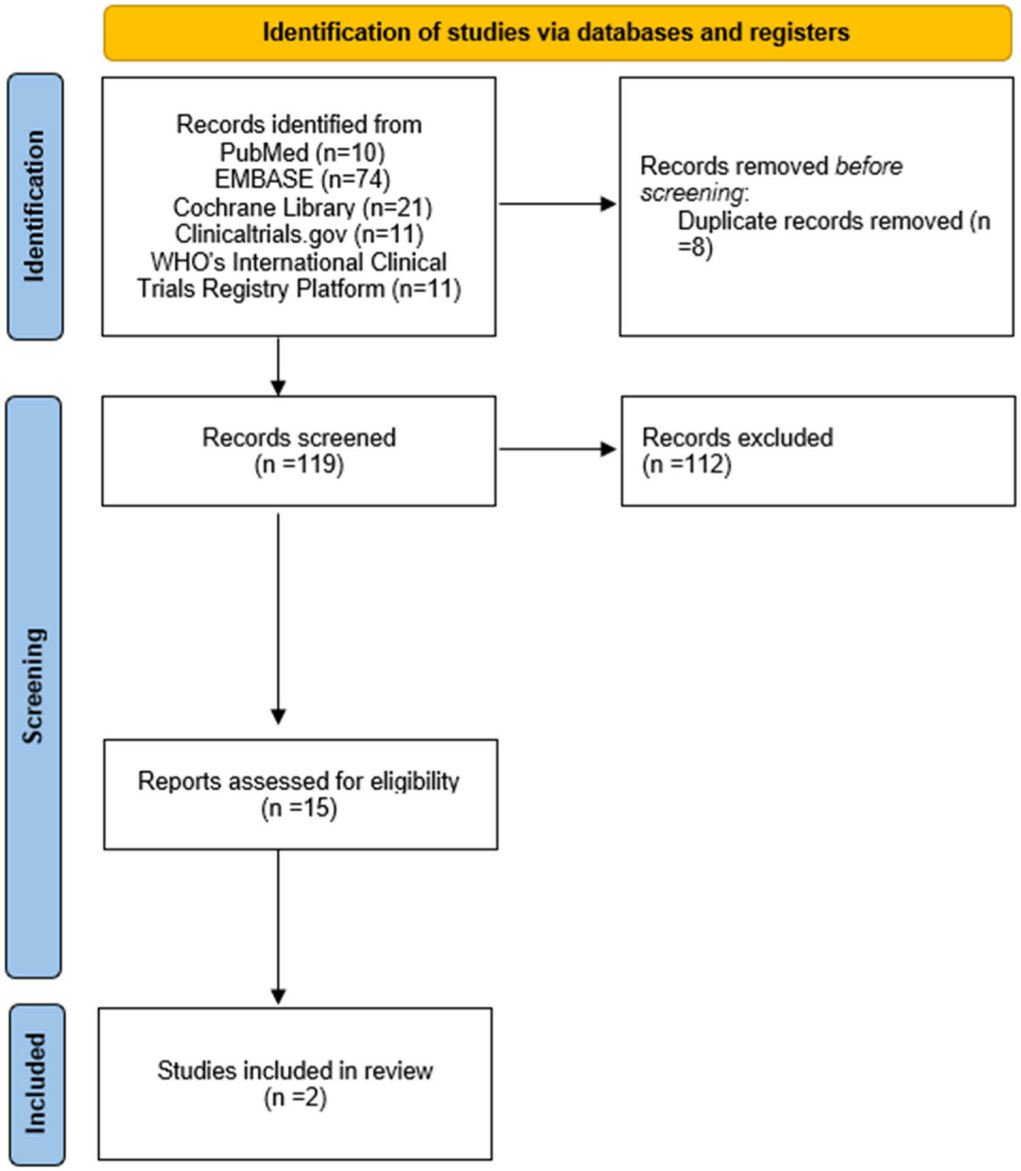
PRISMA flowchart.

The search yielded a total of 127 references and after screening and retrieval, 15 studies were assessed for eligibility. Finally, two RCTs published between 2021 and 2023 (Figure 1),^6^, ^7^ with a total of 237 patients in the Sotatercept group and 192 patients in the Placebo group were included. The primary outcomes of interest were efficacy outcomes including pulmonary vascular resistance (PVR), reduction in N‐terminal pro–B‐type natriuretic peptide (NT‐pro‐BNP) levels, functional improvement of the World Health Organization (WHO) class, and also hemodynamic outcomes including pulmonary artery wedge pressure (PAWP), mean pulmonary artery pressure (Mpap), right atrial pressure (RAP), cardiac output (CO), and change in cardiac index (CI). The secondary outcomes included the incidence of diarrhea, dizziness, fatigue, nausea, headache, thrombocytopenia, adverse events leading to discontinuation or withdrawal, serious adverse events that were deemed by trial investigators, any adverse events reported, and finally an increase in hemoglobin levels.

**Figure 2:**
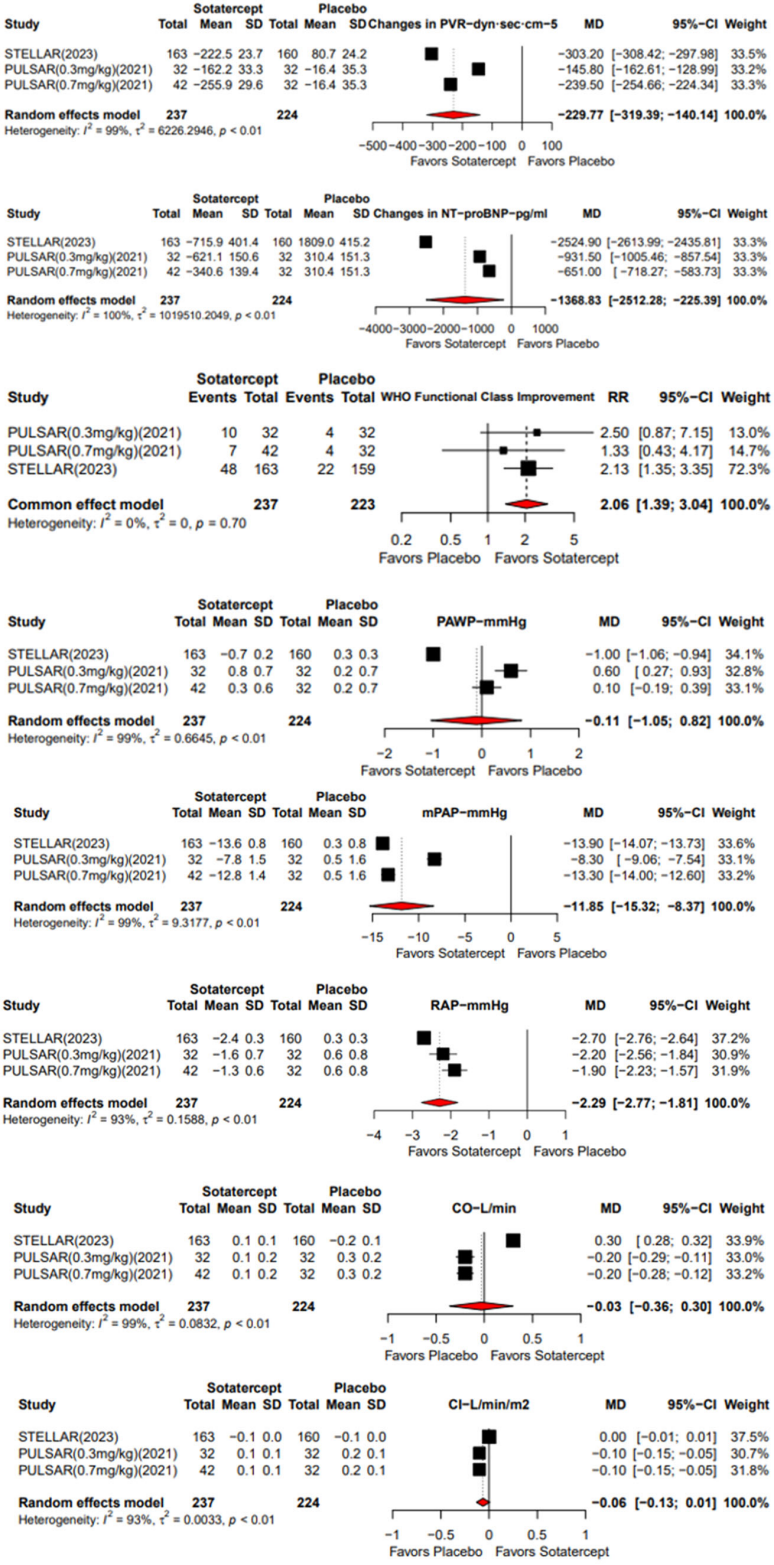
Forest plot showing the observed efficacy outcomes and the estimate of the random effects model for, the change in pulmonary vascular resistance (PVR), N‐terminal pro–B‐type natriuretic peptide (NT‐proBNP), WHO functional class improvement rate, pulmonary artery wedge pressure (PAWP), mean pulmonary artery pressure (Mpap), right atrial pressure (RAP), cardiac output (CO), and change in cardiac index (CI) at Week 24.^6^, ^7^

As depicted in Figure 2, concerning effectiveness outcomes, a pooled analysis demonstrated a notable advantage of Sotatercept over placebo in improving PVR from the starting point, with an MD of −229.77 (95% CI: −319.39; −140.14), improving RAP with an MD of −2.29 (95% CI: −2.77; −1.81), and mPAP with an MD of −11.85 (95% CI: −15.32; −8.37). In contrast, PAWP with an MD of −0.11 (95% CI: −1.05; 0.82), CO with an MD of −0.03 (95% CI: −0.36; 0.30), and CI with an MD of −0.06 (95% CI: −0.13; 0.1) showed minimal changes across the groups.

Improvements from baseline in NT‐proBNP levels with an MD of −1368.83 (95% CI: −2512.28; −225.39) were also noted with Sotatercept.

Additionally, there was a significant improvement in WHO functional performance, with an RR of 2.06 (95% CI: 1.39; 3.04).

Regarding adverse events, pooled RR of diarrhea 1.55 [0.92; 2.61], dizziness 2.70 [0.65; 11.26], and fatigue 0.75 [0.32; 1.76], nausea 0.87 [0.51; 1.47], headache 1.31 [0.88; 1.96], thrombocytopenia 3.37 [1.26; 9.02], an adverse event leading to discontinuation 0.78 [0.12; 5.19], an adverse event leading to withdrawal from the trial 0.48 [0.12; 2.00], serious adverse events 0.94 [0.40; 2.25], and finally any adverse event reported 0.97 [0.90; 1.04] were similar between the Sotatercept and placebo groups, respectively. Moreover, our pooled analysis showed a greater incidence of elevation in hemoglobin levels for the Sotatercept group, with an RR of 11.10 (summary of the results are available in Table 1, and forest plots are shown in Supporting Information: Figure 1) [2.12; 58.18].

**Table 1:** Table showing the observed adverse events rate and the estimate of the random effects model.

Event	RR (95% CI)
Adverse event rate	0.97 [0.90; 1.04]
Diarrhea	1.55 [0.92; 2.61]
Dizziness	2.70 [0.65; 11.26]
Fatigue	0.75 [0.32; 1.76]
Nausea	0.87 [0.51; 1.47]
Headache	1.31 [0.88; 1.96]
Thrombocytopenia	3.37 [1.26; 9.02]
Adverse event leading to discontinuation	0.78 [0.12; 5.19]
Adverse event leading to withdrawal from the trial	0.48 [0.12; 2.00]
Serious adverse events	0.94 [0.40; 2.25]
Increase in Hb−mg/dL levels	11.10 [2.12; 58.18]

## 4 DISCUSSION

Our findings indicate that patients who received Sotatercept showed greater improvement in both PVR and the WHO functional class. According to Maron et al. study, PVR is a crucial predictor of PAH prognosis, and the range of PVR independently predicts significant clinical outcomes such as hospitalizations for heart failure and mortality.^8^ Therefore, given the potential of Sotatercept in reducing PVR, it may offer a preferred approach to treating PAH. Additionally, the incidence of adverse events was similar between the Sotatercept and placebo groups. Moreover, the number of cases with an increase in hemoglobin levels was significantly higher in the Sotatercept group. However, due to the fact that around 33% of patients with pulmonary hypertension suffer from anemia,^9^ and both anemia and polycythemia can have adverse effects on the disease's prognosis,^9^, ^10^ further investigation is required to assess the potential advantages and drawbacks of utilizing Sotatercept as a treatment option with regard to its erythropoietic effects. It is essential to manage anemia and polycythemia effectively to enhance outcomes for individuals with PAH. Concerning long‐term effectiveness and safety, only the interim results from the PULSAR open‐label extension have been published, indicating that clinical efficacy is sustained across the study for up to 48 weeks. Additionally, safety was in line with earlier findings in PAH and other patient groups.^11^ However, further follow‐up data on the outcomes of patients receiving Sotatercept treatment are undoubtedly required. This information may aid in the development of future clinical trials and inform treatment decisions for patients with different PAH subgroups.

## 5 LIMITATIONS

We acknowledge that our research may have some limitations. Specifically, there have been only two RCTs conducted to evaluate the effectiveness and potential side effects of Sotatercept, which could impact the overall accuracy of our pooled analysis. Furthermore, only one study provided information on the extended follow‐up of Sotatercept in treating PAH, making it impossible to compare the long‐term outcomes and adverse events between the Sotatercept and Placebo groups. Also, while subgroup analyses can provide valuable insights into potential variations in outcomes, our study was constrained by the limited number of studies and data points within certain subgroups.

## 6 CONCLUSION

Sotatercept could be a hopeful drug for individuals with PAH. It has demonstrated an improvement in PVR and WHO functional class, although an increase in hemoglobin levels was seen without a significant increase in any other adverse events. Nevertheless, more research is necessary to examine the potential advantages and disadvantages of its erythropoietic effects, particularly in patients with anemia or polycythemia. Furthermore, additional long‐term data is needed to evaluate the safety and efficacy of Sotatercept in treating PAH.

## AUTHOR CONTRIBUTIONS

*Conceptualization*: Amir Nasrollahizadeh and Hamidreza Soleimani. *Methodology*: Ali Nasrollahizadeh. *Software*: Hamidreza Soleimani. *Validation*: Hamidreza Soleimani, Ali Nasrollahizadeh, and Amir Nasrollahizadeh. *Formal analysis*: Hamidreza Soleimani. Investigation: *Amir Nasrollahizadeh*. *Resources*: Ali Nasrollahizadeh. *Data curation*: Hamidreza Soleimani. *Writing—original draft preparation*: Amir Nasrollahizadeh. *Writing—review and editing*: Ali Nasrollahizadeh. *Visualization*: Kaveh Hosseini. *Supervision*: Kaveh Hosseini. *Project administration*: Amir Nasrollahizadeh. *Funding acquisition*: Kaveh Hosseini. All authors have read and agreed to the published version of the manuscript.

## CONFLICT OF INTEREST STATEMENT

The authors declare no conflict of interest.

## Supporting information

Supporting information.

## Data Availability

The data set is not publicly available. Requests to access these data sets should be directed to the corresponding author, Kaveh_hosseini130@yahoo.com.
