# Supplementary material for: Navigating the Sotatercept landscape: A meta‐analysis of clinical outcomes
Source: Clin Cardiol. 2023 Oct 11;47(1):e24173. doi: 10.1002/clc.24173 (PMC10766119; doi:10.1002/clc.24173)

Supplementary Table 1. Search strategy

| PubMed | "sotatercept AND pulmonary arterial hypertension [MESH]” |
| --- | --- |
| Embase | sotatercept AND pulmonary arterial hypertension [Emtree terms]" |

Supplementary figure 1. Forest plot showing the observed adverse events rate and the estimate of the random effects model.
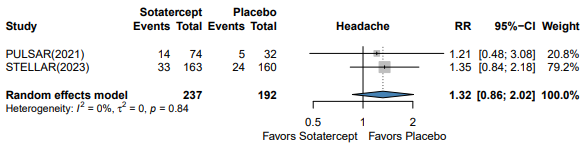

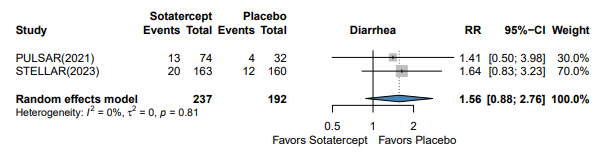

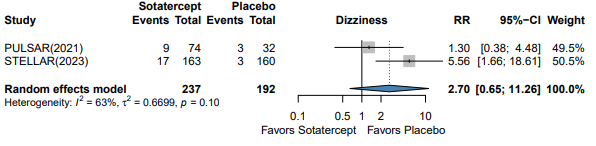

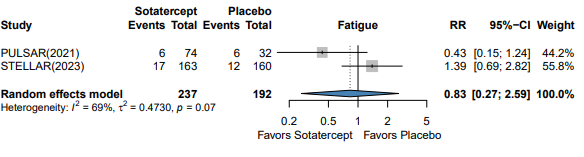

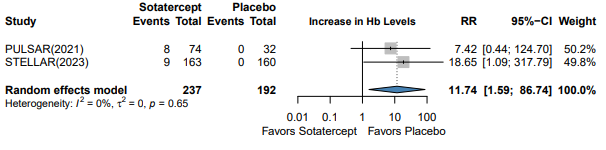


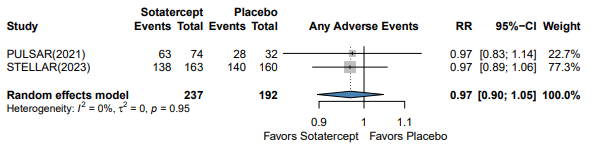


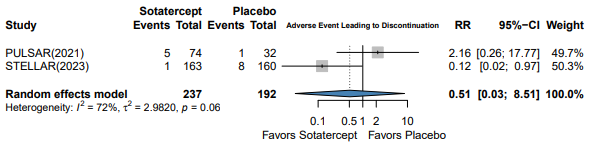


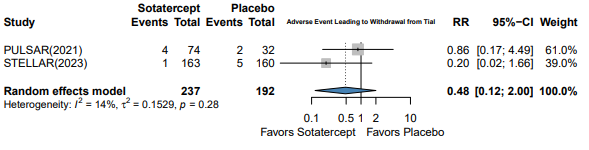


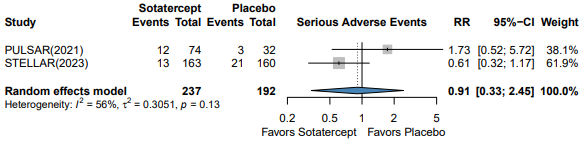


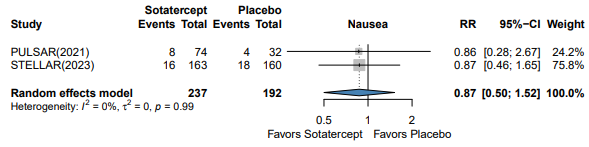

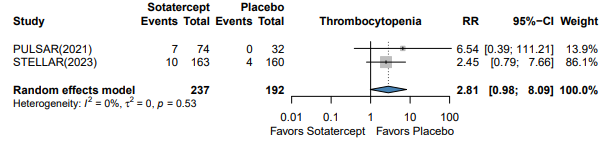

Supplement: Supplementary file 1 — Supporting information. [file CLC-47-e24173-s001.docx]
